# Cytogenetic characterization and *H-ras *associated transformation of immortalized human mammary epithelial cells

**DOI:** 10.1186/1475-2867-6-15

**Published:** 2006-05-26

**Authors:** Krishna Rao, Özge Alper, Kent E Opheim, George Bonnet, Kristine Wolfe, Eileen Bryant, Siobhan O'Hara Larivee, Peggy Porter, James K McDougall

**Affiliations:** 1 Southern Illinois University School of Medicine Cancer Institute, P.O. Box 19678, Springfield, IL 62794-9678, USA; 2 National Institutes of Health, National Institute of Neurological Disorders, Surgical Neurology, Bldg 10, 5D37, Bethesda, MD 20892-1414, USA; 3 Children's Hospital and Regional Medical Center, Dept. of Laboratories, A-6901 4800 Sand Point Way, NE, Seattle, WA 98105, USA; 4 Cytogenetics Studio, Inc., 41 Myrtle Ave., Cambridge, MA 02138, USA; 5 2511 Boundary St., San Diego, CA 92104-5313, USA; 6 Fred Hutchinson Cancer Research Center, Clinical Research Division, P.O. Box 19024, 1100 Fairview Avenue North, D2-190, Seattle, WA 98109-1024, USA; 7 Seattle Cancer Care Alliance, 825 Eastlake Avenue East, Mailstop G7500, Seattle, WA 98109, USA; 8 Fred Hutchinson Cancer Research Center, Cancer Biology Program, P.O. Box 19024, 1100 Fairview Avenue North, C1-015, Seattle, WA, 98109-1024, USA; 9 Department of Pathology, University of Washington School of Medicine, Seattle, WA 98195, USA

## Abstract

**Introduction:**

Immortalization is a key step in malignant transformation, but immortalization alone is insufficient for transformation. Human mammary epithelial cell (HMEC) transformation is a complex process that requires additional genetic changes beyond immortalization and can be accomplished in vitro by accumulation of genetic changes and expression of H-ras.

**Methods:**

HMEC were immortalized by serial passaging and transduction with the catalytic subunit of the human telomerase gene (*hTERT*). The immortalized cells were passaged in vitro and studied by a combination of G- banding and Spectral Karyotyping (SKY). H-ras transduced, hTERT immortalized cells were cloned in soft agar and injected into nude mice. Extensive analysis was performed on the tumors that developed in nude mice, including immunohistochemistry and western blotting.

**Results:**

Immortal HMEC alone were not tumorigenic in γ-irradiated nude mice and could not grow in soft agar. Late passage hTERT immortalized HMEC from a donor transduced with a retroviral vector containing the mutant, autoactive, human H-*ras*61L gene acquired anchorage independent growth properties and the capacity for tumorigenic growth *in vivo*. The tumors that developed in the nude mice were poorly differentiated epithelial carcinomas that continued to overexpress ras. These cells were resistant to doxorubicin mediated G1/S phase arrest but were sensitive to treatment with a farnesyltransferase inhibitor.

**Conclusion:**

Some of the cytogenetic changes are similar to what is observed in premalignant and malignant breast lesions. Despite these changes, late passage immortal HMEC are not tumorigenic and could only be transformed with overexpression of a mutant H-ras oncogene.

## Introduction

Immortalization is a key step in malignant transformation, as it allows a cell to indefinitely proliferate while accumulating genetic abnormalities. Normal human mammary epithelial cells (HMEC) initially proliferate robustly in tissue culture before encountering a proliferation block termed stasis [1,2]. Cells in stasis are less proliferative, appear large and flattened, stain positively for beta galactosidase, but are non-apoptotic since the cells are negative by terminal deoxynucleotidyl transferase mediated dUTP nick end labeling (TUNEL) staining. HMEC can escape stasis spontaneously, as evidenced by the fact that a small percentage of HMEC in culture may pass through stasis and continue to proliferate [1]. The exact number of passages and percentage of cells that evade this initial growth arrest are highly dependent upon individual specimens and culture conditions [1]. It has been demonstrated that the p16 ^INK4A ^tumor suppressor is inactivated in HMEC escaping stasis [3], primarily by progressive promoter methylation [4-6]. Cells undergo a second mortality stage, termed agonescence [7]. Cells in agonescence appear small and vacuolated and have critically shortened telomeres. Activation of telomerase expression at this stage stabilizes telomere length, allowing cells to pass through agonescence and proliferate indefinitely.

DNA tumor viruses immortalize cells and provide a useful model for studying neoplasia. For example, the combined expression of the catalytic subunit of human telomerase (*hTERT*), together with SV40 large T antigen can immortalize HMEC. The E6/E7 gene products from "high risk" human papillomavirus types such as HPV16 can uniquely immortalize HMEC at any stage without the aid of *hTERT *[8].

*In vitro *models of mammary cell transformation either require the introduction of viral oncogenes [9-11] or make use of chemical carcinogens [12], such as benzo [a]pyrene. Stampfer and Bartley [12] exposed rapidly growing primary cultures of human mammary epithelial cells to benzo [a]pyrene for 2–3 days and produced two immortalized cell lines- 184 A1 and 184 B5. The introduction of *Ras *or *erbB2 *into subline 184A1N4 or 184B5 respectively results in production of a malignant phenotype [12,13]. Both 184 A1 and 184 B5 have acquired multiple cytogenetic abnormalities [14]. HBL 100, a cell line derived from an immortalized mammary cell cultured from mother's milk, also transforms with the introduction of a single oncogene [15], but carries an integrated copy of the SV40 genome and demonstrates a near triploid karyotype.

More recently, Elenbaas et al. [9] transformed HMEC by serial transduction of SV40 large T antigen, SV40 small t antigen, *hTERT*, and a mutant, constitutively active, human V12G H-*ras*. Substantial overexpression of H-ras (12 fold) was required in order to transform the HMEC. The tumors that were obtained were epithelial, staining positively for cytokeratin, and negative for the estrogen and progesterone receptor, consistent with the poorly differentiated histology seen on hematoxylin and eosin staining. Cytogenetic data presented in the paper revealed frequent increased copy number of the locus containing the c-Myc oncogene, either through trisomy 8, translocations involving 8q24, or by creation of isochromosome for the long arm of chromosome 8 [i(8)(q10)]. Interestingly, cells in the tumor, in which i(8)(q10) was frequently seen, displayed extra copies of chromosomes 5. One tumor had 22% metaphases with del(12p). One of the tumors was near tetraploid while the other two were near diploid (90% and 62% of total cells analyzed).

Toouli et al. [11] transformed HMEC from a single donor purely through the use of SV40 large T antigen and SV40 small t antigen. Fifty percent of the late passage cell lines expressing both large T antigen and small t antigen were tumorigenic. One of the lines showed a 30% take rate, with a latency of twenty-one days, while the other line showed a 20% take rate with a latency of sixty-eight days. No histologic, cytogenetic, or immunohistochemical data were presented on the tumors. The authors also noted that none of the hTERT immortalized HMEC lines were tumorigenic.

In this paper, we describe the cytogenetic characteristics of a hTERT immortalized human mammary epithelial cell line, and of a tumorigenic human mammary epithelial cell line produced through hTERT immortalization, passaging, transduction of H-*ras*61L, and soft agar cloning.

## Materials and methods

### Isolation and culture of HMEC

The mammary epithelial cells (HMEC) were all derived from reduction mammoplasties of individuals with no known breast pathology, as reconfirmed by histopathology of the post-surgical specimens. HMEC were prepared by the method of Smith et al. [16] and grown at 37°C and 5% CO_2 _in DFCI-1 media [17]. Cells were allowed to pass through stasis before infection with the *hTERT *construct, as described by Foster et al.[18], Kiyono et al.[3], and Rothenberg and Nolan [19].

### Cell proliferation and staining

HMEC (human normal mammary epithelial cells) and our tumorigenic cells were seeded in glass 8-well chamber slides at a concentration of 10^4 ^cells per well and treated with FTI-277 and DMSO at 2.5, 5.0, 10.0, 15.0 and 20.0 μM concentrations for 5 days in duplicate. Cells were counted using Hemocytometer (blood cell counter) on 1^st^, 2^nd ^and 5^th ^days as well as stained with Phalloidin-TXR for cytoskeletal staining and DAPI for nuclear staining.

### Cytogenetics

Metaphase chromosomes from HMEC were prepared and G-banded (Wright's stain) using standard cytogenetic techniques [20]. Briefly, after the cells were incubated with 0.06 ug/ul of colcemid at 37°C for 2.5 hours, they were trypsinized with trypsin/EDTA (0.5%/0.1%) and incubated in 2 ml of a 1:1 (by vol) mixture of 0.8% sodium citrate and 0.075 M potassium chloride for thirty minutes at 37 degrees C. This was followed by addition of 1 ml of Carnoy's fixative (3:1 by vol; methanol:acetic acid) and incubation at room temperature. The fixed cells were then spun down and resuspended in 1 ml of Carnoy's fixative. Metaphase chromosomes were prepared for G-banding and Spectral Karyotyping (SKY). Cells were analyzed based on clonality criteria and karyotypes were described by the recommendations of the International System for Human Cytogenetic Nomenclature (ISCN 2005) [21].

### Spectral karyotyping

SKY [22] was used to identify or confirm the G-band karyotype assignments. Briefly, SKY is a form of fluorescence in situ hybridization (FISH) which utilizes a mixture of "whole chromosome paint" DNA probes to uniquely label each of the 24 different human chromosomes, allowing simultaneous visualization and identification of each chromosome or chromosome derivative. The limit of resolution of SKY for structural abnormalities is currently unknown, and the technique will likely not detect subtle, intra-chromosomal rearrangements, such as small deletions, duplications or inversions. We also cannot rule out the possibility of mosaicism for numerical or structural abnormalities given the limited number of cells (usually five), which are evaluated in this study. Unbanded or de-stained G-banded metaphase chromosome preparations were hybridized with the SKY probe mixture and were analyzed using SKYView imaging software, following the manufacturer's instructions (Applied Spectral Imaging, Inc.; Carlsbad, CA). The hybridized metaphase cells were simultaneously stained with the nuclear stain, DAPI. When the bright-dark fluorescent bands from chromosomes stained with DAPI are reversed by the SKYView imaging software, a low-resolution G-band-like pattern is produced that is used to confirm the chromosome identity and to assign cytogenetic breakpoints on rearranged chromosomes.

For both G-band and SKY analyses, structural chromosome abnormalities and chromosome gains were considered clonal if the aberration was exhibited in at least two cells from a culture. Whole chromosome losses were considered clonal if observed in at least three cells from a culture, per ISCN convention [21].

### Flow cytometry

The protocol as previously described was followed [23]. Cells were lifted from the plate with trypsin/EDTA (0.5%/0.1%) and fixed overnight in 80% ethanol at 4°C. Cells were washed with PBS the following day, and this was followed by staining in PBS with 10 ug/ml of propidium iodide for twenty minutes at room temperature.

### Transduction of H-ras 61L into HMEC

pCTV3H H-*ras*61L (a generous gift of Dr. Channing Der) or pCTV3H (empty control vector) was precipitated and incubated with Phoenix cells to produce infectious supernatant [19]. Cells were transduced with either construct by incubation with 1 ml of infectious supernatant with 4 pg/ml of polybrene for four hours. Two days later, continuous selection with hygromycin B (20 ug/ml) was initiated over a several week time span until sustained growth from a robust population was maintained.

### Soft agar assay

Six-well plates were filled with two ml of 0.66% noble agar in DFCI-1 media as a bottom layer and allowed to solidify at 4°C overnight. 10^5 ^cells were suspended in one ml of 0.33% noble agar in DFCI-1 media and plated onto wells. Each well was fed once a week with one ml of 0.33% agar in DFCI-1 media. Wells were scored for colony growth over the next four weeks. Cell clumps greater than 60 um were scored as colonies.

### Nude mice injection and tumor harvesting

Four-week-old female nude mice were γ-irradiated to 300 rads using a linear accelerator and injected subcutaneously over the abdomen with 10^7 ^cells derived from the soft agar colony that was regrown in monolayer. The mice were observed for tumor formation over the following 30 days. Tumors were harvested and cut into two parts. One part was sent for paraffin embedding, while the other half was minced and disaggregated with trypsin/EDTA (0.5%/0.1%) overnight at 4°C, followed by trypsinization for an additional thirty minutes at 37°C. Single cells were plated and allowed to grow in DFCI-1 media.

### Immunohistochemistry

Immunohistochemistry on paraffin embedded tumor sections was performed as previously described by Gown and Vogel [24] at Phenopath Laboratories (Seattle, WA) or the laboratory of Dr. Peggy Porter (Program in Cancer Biology, Fred Hutchinson Cancer Research Center, Seattle, WA) using 4 um thick sections baked onto positively charged slides. Immunohistochemical studies were performed on formalin-fixed, paraffin-embedded, tissue sections using the avidin-biotin-peroxidase complex (ABC) method. Sections were incubated with a cocktail of two anti- cytokeratin mouse monoclonal antibodies (AE1 and AE3, Roche, Indianapolis, IN, 1:200 dilution) that recognizes a wide range of high and low molecular weight cytokeratins; anti-cytokeratin-7 (OV-TL 12/30, DAKO, Carpinteria, CA, 1:1000), anti-smooth muscle myosin heavy chain (SMMS-1, DAKO, 1:30); anti-estrogen receptor (ID5, Zymed, South San Francisco, CA, 1:200); anti-progesterone receptor (IA6, Lab Vision, 1:50); anti-mammoglobin(304-1A5 1;2000, 31A5 1:400, Corixa, Seattle, WA); anti-cytokeratin 5/6(5/16 B4, Chemicon, Temecula, CA, 1:1000); anti-cytokeratin 19 (BA 17, DAKO, 1:100), anti-calponin (Calp, DAKO, 1;1000); anti-cytokeratin 20 (KS 20.8, DAKO, 1:250); anti-cytokeratin 8 (35βH11, DAKO, 1:50); anti-E-cadherin (HECD-1, Zymed, San Francisco, CA,1:300); brst-2 (D6, Signet, Dedham, MA, 1:200). After overnight incubation, slides were incubated with secondary antibody. For polyclonal antibodies raised in rabbit, biotinylated goat anti-rabbit immunoglobulin (1:200) was used. For murine monoclonal antibodies, we used biotinylated goat anti-mouse immunoglobulin (1:200). Sections were incubated in secondary antibody for 30 minutes. The peroxidase staining procedure was done using the ABC Elite kit (Vector laboratories, Burlingame, CA). The immunostaining reaction was visualized using 0.05 % 3,3'- diaminobenzidine in Tris- HCl buffer containing 0.01 % hydrogen peroxide, pH 7.6.

### Western blotting

Total cell protein was extracted from transduced cells by sonication in We 16 buffer (25 mM Tris HCl- pH 7.5, 125 mM NaCl, 2.5 mM EDTA, 0.05% SDS, 0.5% NP40, 0.5% DOC, 10% glycerol, 1 mM DTT, 0.5 mg/ml Pefablock, 0.025 mg/ml leupeptin, 0.01 mg/ml pepstatin, 0.01 mg/ml aprotinin, 0.08 M β glycerophosphate, 0.005 Na_3_VO_4_, 0.05 M NaF). Twenty micrograms of protein from each cell population was electrophoresed and transferred to a polyvinylidene difluoride membrane (NEN). The membranes were blotted with the following antibodies: H-ras (LA-069, Quality Biotech, Camden, NJ, 1:10,000); c-Myc (9E10, BD Biosciences, San Diego, CA, 1:250), Mdm2 (IF2, Oncogene Research Products, Boston, MA, 1:50), p53 (DO-1, Oncogene Research Products, 1:100), and actin (c-11, Santa Cruz Biotechnology, Santa Cruz, CA, 1:100). Samples destined for H-ras western blotting were run on a 15% SDS polyacrylamide gel, while all other samples were run on 8% polyacrylamide gel. After the samples were transferred onto the membrane, the membrane was blocked in 10% milk in TBST buffer (10 mM Tris-HCl, pH 7.5, 150 mM NaCl, 0.05% Tween-20) for one hour at room temperature or overnight at 4°C. Membranes were then incubated overnight at 4°C with 10 ml of 5% milk in TBST/0.5% Tween 20 with the primary antibody. The following day, membranes were briefly washed and incubated for one hour with secondary antibody in 5% milk in TBST/0.5% Tween 20 with the appropriate secondary antibody. Anti- mouse IgG- HRP from Jackson Immunoresearch Labs (Bar Harbor, ME) was used against the H-ras (1:35,000), c-Myc (1:10,000), and p53 (1:10,000) primaries. Donkey anti-goat antibody (Santa Cruz Biotechnology) was used against the polyclonal actin primary (1:5,000). Blots were developed using the Renaissance chemiluminescence system (NEN) and imaged on Kodak Xomat blue film.

## Results

hTERT immortalized HMEC from donor # 4 (herein referred to as HMEC 4-hTERT or RAO-1) at late passage demonstrated a derivative chromosome 1, [der(1)t(1;8)(p13;q13)], in addition to two normal copies of chromosome 1 and two normal copies of chromosome 8, in four of five metaphase cells analyzed by SKY. HMEC 4-hTERT demonstrated trisomy of 1q and 8q due to the presence of the derivative chromosome, and showed complete trisomy of chromosomes 5 and 20. Flow cytometry of HMEC 4-hTERT confirmed a near diploid population. These immortal cell lines analyzed were not tumorigenic in nude mice and did not demonstrate anchorage independent growth in soft agar.

Transduction of pCTV3H H-*ras*61L into hTERT-immortalized late passage (passage 32) HMEC from donor 4 (RAO-2 cell population) resulted in high levels (at least six fold) of H-ras expression in these cells as compared to HMEC (passage 43). At passage 43, 10^5 ^cells were cast into soft agar. H-ras-transduced HMEC demonstrated growth in soft agar, with at least fifty colonies generated. Of the three colonies picked at random, the second colony was expanded in monolayer and injected, at passage 47, into six nude mice, producing mammary epithelial carcinomas (RAO-4 cell line). The other two colonies, when injected into nude mice, produced spindle cell carcinomas (RAO-3 cell line) and have been described separately [25].

**Figure 1:**
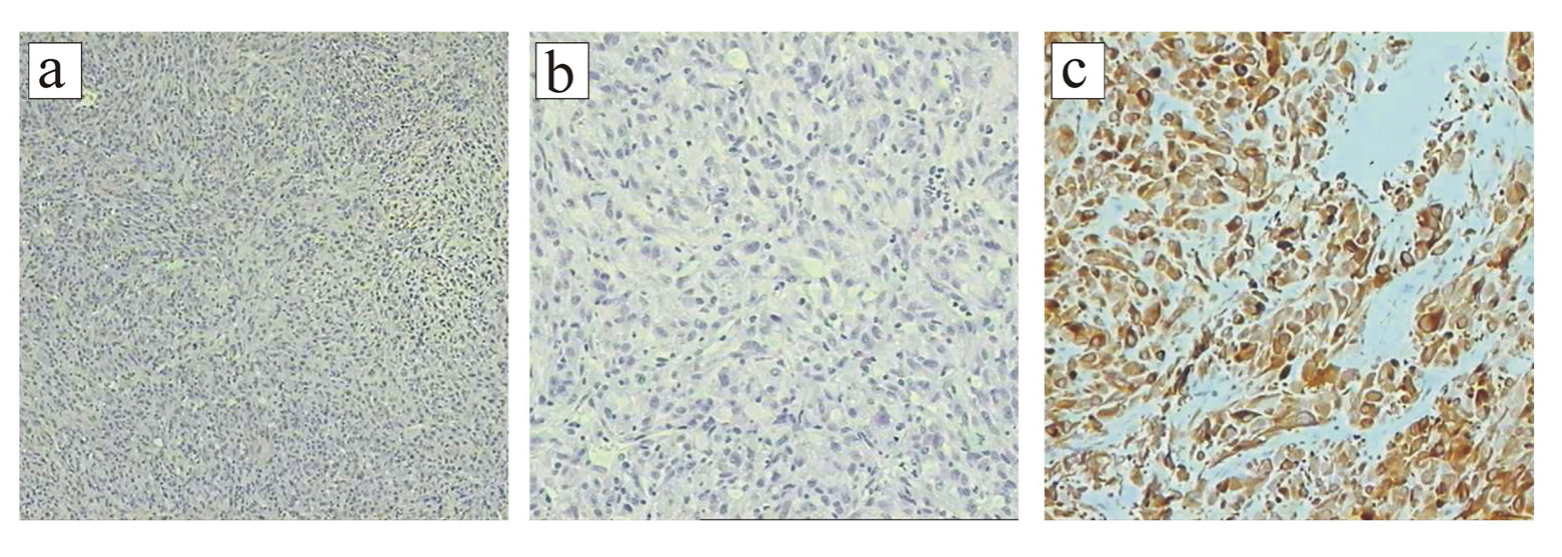
a. Photomicrograph displays a hematoxylin/eosin section (of a section of the mammary epithelial tumor at 40×). b. This same section at 200×. c. The pan-cytokeratin stain (AE1/AE3) is strongly positive, confirming the epithelial nature of the tumor. Magnification is 200×.

All six mice injected with cells from colony 2 of H-*ras*61L transduced HMEC developed tumors subcutaneously at the site of injection within 21 days. Figures 1a and 1b illustrate the hematoxylin/eosin staining of a paraffin-embedded section of the epithelial tumor. The cells are round and circumscribed. The cells form a sheet with no differentiated structure and display areas of necrosis, all consistent with a poorly differentiated carcinoma. The pan-cytokeratin staining (Figure 1c) is strongly positive, consistent with the tumor's epithelial origin. The tumor is cytokeratin 7 positive and negative for cytokeratins 8, 18, and 20. It is negative for basal/myoepithelial markers such as cytokeratin 5, smooth muscle myosin heavy chain, and calponin. The tumor is negative for the estrogen receptor and the progesterone receptor. It is negative for mammoglobin and the brst-2 antigen. The tumor has also lost E-cadherin expression (data not shown).

**Figure 2:**
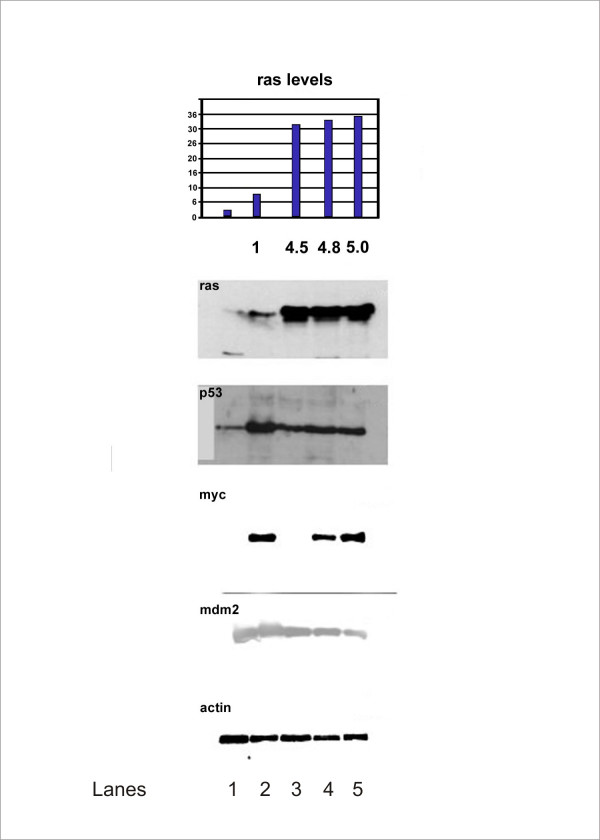
Western blotting (confirmed from three separate experiments) of human mammary epithelial cells (HMEC) from donor 4 at various stages from mortal, early passage to the epithelial tumor are shown below. Volume intensity of H-ras is displayed on the bar graph at the top, and H-ras overexpression compared to the baseline mortal state is indicated below each bar. Lanes (from right to left): 1. HMEC from donor 4- post stasis, pre agonescence, passage 9. 2. RAO-1 cells. 3. RAO-2 cells. 4. Cells from soft agar colony that developed into the epithelial tumor in nude mice. 5. RAO-4 cells, cultured in monolayer

Western blot analysis of HMEC from donor 4 reveals some interesting patterns, shown in Figure 2. H-ras levels are very low in early passage HMEC. With immortalization and subsequent passaging, H-ras levels increase. After transduction of pCTV3 H-*ras*61L, H-ras levels increase substantially and are maintained at a five-fold level of overexpression through to the tumor. Expression of p53 was at low levels in mortal cells. In late passage immortal cells, it is expressed at high levels. Ras transduction apparently reduces the level of p53 expression, and a reduced level of p53 expression is observed in the tumor. c-Myc takes a very interesting pattern of expression. c-Myc is at low levels in our post-selection HMEC (lane 1, Figure 2). It is strongly expressed in RAO-1 (lane 2, Figure 2), but diminished in RAO-2 cells (lane 3, Figure 2), suggesting that H-*ras*61L transduction reduces c-Myc expression. Finally, c-Myc expression is detected in the soft agar colony (lane 4, Figure 2) that gave rise to the mammary epithelial tumor, and in the RAO-4 tumor cells cultured in monolayer (lane 5, Figure 2). Mdm 2 levels are stable throughout this time course.

**Figure 3:**
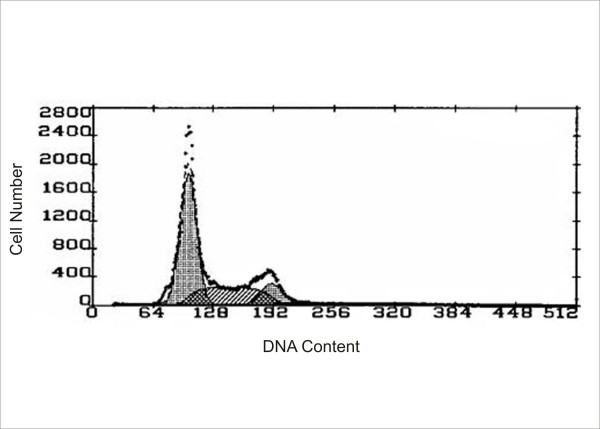
Flow cytometry data is represented below. There is no tetraploid population noted.

**Figure 4:**
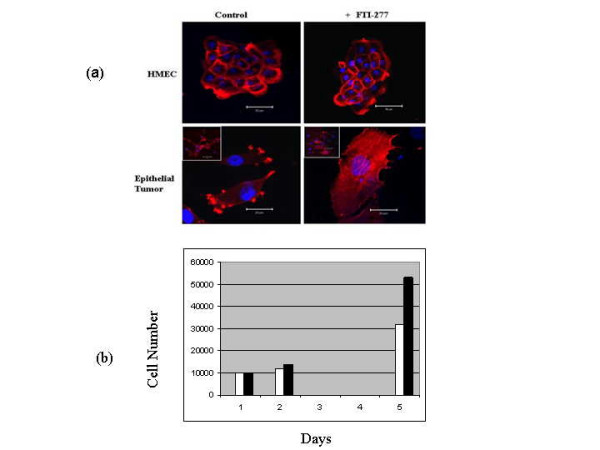
(a) Primary HMEC and RAO-4 cells were treated with FTI-277, a potent farnesyl transferase inhibitor, and effects were observed. The membranous ruffles of cells showed a significant flattened morphological appearance after treatment with FTI-277 at 2.5 and 5.0 μM concentrations. Although cellular morphological changes were not observed in primary HMEC at these concentrations, we observed some cell death with FTI-277 treatment at 2.5 and 5.0 μM concentrations. (b) We found a reduction in RAO-4 cell proliferation with FTI-277 treatment at 5.0 μM concentrations. The black bars represent untreated epithelial tumor cells while the white bars represents treated epithelial tumor cells.

Single color (DAPI) flow cytometry (Figure 3) of mammary epithelial tumor cells revealed a near diploid population with no significant tetraploid population, confirming the results of cytogenetic analysis. The treatment of H-ras transduced cells with FTI-277, a farnesyl transferase inhibitor, resulted in a significant change in cell phenotype observed by a flattened membraneous morphology in (Figure 4a) and a decrease in cell number over a period of five days (figure 4b), suggesting that the cell line is quite sensitive to ras inhibition. No cellular morphological changes were observed in HMEC at these concentrations.

**Figure 5:**
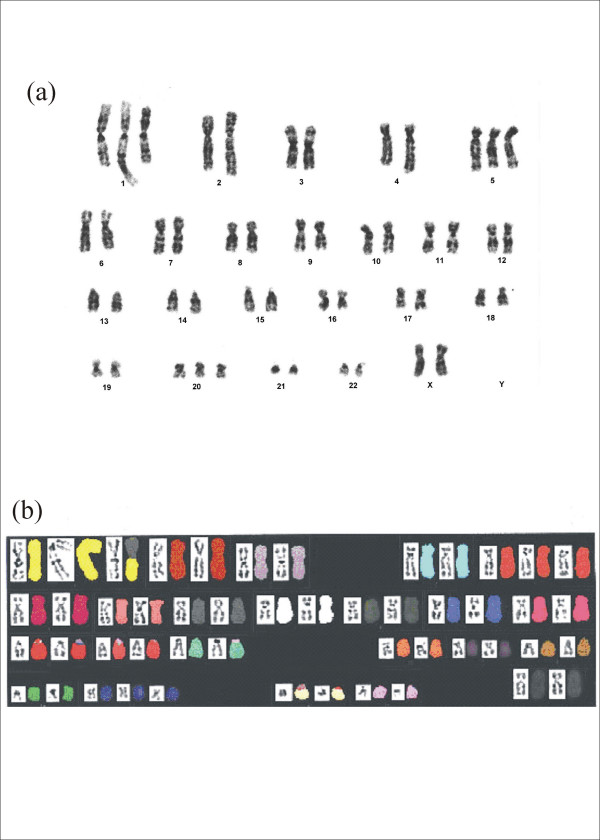
(a) A representative G-banded karyotype of the mammary epithelial tumor is seen below. The mainline karyotype is 49,XX,+der(1)t(1;8)(p13;q13),psu idic (1)(q23),+5,+20. (b) Spectral Karyotype of the cells from the mammary epithelial tumor confirms the G-banded cytogenetic analysis.

**Figure 6:**
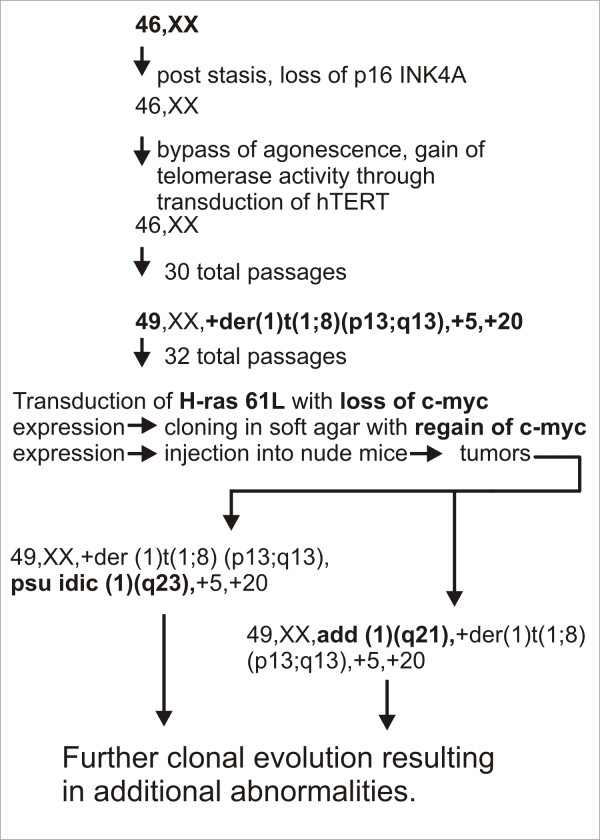
Summary of karyotypic changes from early passaged HMEC towards mammary epithelial carcinoma. The mainline karyotype of the tumor and stepwise cytogenetic changes are highlighted in bold face type.

Cytogenetic analysis of the tumor revealed at least two related cell populations: 49, XX, +der(1)t(1;8)(p13;q13), psu idic(1)(q23),+5,+20 (Figure 5a; Figure 6) and 49, XX, add(1)(q21),+der(1)t(1;8)(p13;q13),+5,+20 (Figure 6). Among the cells analyzed, there were chromosome losses, and analysis could not determine whether they were clonal or non-clonal.

Spectral Karyotyping was performed on late passage hTERT immortalized cells (prior to Hras61L transduction) from donor 4, confirming the presence of many of the cytogenetic abnormalities seen in the epithelial tumor. The mainline karyotype in these cells was 49, XX, +der(1)t(1;8)(p13;q13), +5, +20. Despite the presence of these extensive cytogenetic abnormalities, these cells were non-tumorigenic in gamma-irradiated nude mice. Spectral Karyotyping of the epithelial tumor (Figure 5b) revealed the mainline karyotype to be 49, XX,+der(1)t(1;8)(p13;q13), psu idic(1)(q23), +5, +20.

## Discussion

We hypothesize that the HMEC studied here escaped a telomere length associated crisis through early passage transduction with hTERT resulting in stabilization of telomeres and transient maintenance of a diploid state, as well as immortalization. However, the constitutive expression of the catalytic subunit of telomerase through agonescence and the disruption of the Rb pathway may carry a price, as the immortalized cells studied here ultimately developed specific chromosomal abnormalities (trisomy 5, trisomy 20, gain of 8q, and abnormalities of chromosome 1, some of which are seen in breast cancer and other neoplasias. Artandi et al. [26] demonstrated that mTERT transgenic FVB mice displayed a statistically significant susceptibility to breast cancer. This murine strain is susceptible to develop a variety of tumors such as lung adenocarcinomas, lymphomas, and sarcomas but is resistant to developing spontaneous breast carcinomas. The mTERT transgenic FVB murine mammary tumors exhibited diverse histological patterns. Additionally, preinvasive lesions were noted in anatomically separate regions of the mammary gland, suggesting that constitutive mTERT expression led to the promotion of preneoplastic lesions. The authors concluded that telomerase may have dual roles of stabilizing telomeres and have a second role that does not depend on the setting of limited telomere reserve. Mad 2 is expressed at aberrantly high levels throughout the cell cycle in cells with Rb pathway defects. Hernando et al. [27] demonstrated that partial suppression of Mad2 expression to nearly normal levels in immortalized cells resulted in a reduced aneuploid fraction. The authors suggest that aberrant expression of Mad2 results in a hyperactive spindle checkpoint, altering the sequence of mitotic events and the fidelity of chromosome transmission. hTERT immortalized HMEC 4 demonstrated consistent gain of chromosomes 5, 20, 1q, and 8q. c-Myc, which is located on 8q24, is a potential candidate gene for copy number increase in these cells.

Based on the keratin distribution in the normal mammary gland [28], the epithelial tumor generated in this study represents a poorly differentiated malignancy derived from a lobular, luminal cell. This phenotype most likely appears after selection in soft agar as the preceding cells do express luminal markers. The epithelial tumor strongly expresses cytokeratin 7, which is found predominantly in luminal cells. It is negative for cytokeratin 20, consistent with its mammary origins. It is also negative for cytokeratins 8 and 18, which are found predominantly in the ductal cells. It completely lacks cytokeratin 5, smooth muscle myosin heavy chain, and calponin, which excludes derivation from the basal and myoepithelial compartments. Finally, the tumor is negative for Estrogen receptor, Progesterone receptor, brst-2 antigen, and mammoglobin. Brst-2 antigen is found predominantly in mammary tumors with elements of apocrine differentiation while mammoglobin is found in a minority of mammary tumors [29].

The tumor is negative for E-cadherin staining, further reinforcing its putative lobular origin. Acs et al. [30] reviewed the E-cadherin expression in 183 invasive mammary carcinomas and 198 carcinomas in situ and found that moderate to strong staining was present in all invasive and in situ ductal carcinomas while virtually all in situ and invasive lobular carcinomas lost expression of E-cadherin. Droufakou et al. [31] analyzed 22 invasive lobular carcinomas and demonstrated several different mechanisms for loss of E-cadherin expression including promoter methylation, mutation, or loss of heterozygosity in any combination. Methylation may also play a key role in silencing E-cadherin expression in the tumor generated in this study. As noted previously, the HMEC in this study were transduced with mutant H-*ras*, cloned in soft agar, and injected into nude mice. During this process, c-Myc expression was reduced after passaging and transduction, but apparently regained during soft agar cloning. The re-expression of myc may be critical to the transformation of HMEC. The oncogenic potency of the myc/ras combination has been known for quite some time [32]. The levels of c-Myc and p53 rise with repeated passaging [11,33] in hTERT immortalized HMEC. However, they are reduced quite dramatically following H-*ras*61L transduction. Because c-myc expression is detected in the soft agar colony that gave rise to the epithelial tumor, as well as in the tumor itself, we hypothesize that c-myc expression plays an important role in mammary cell transformation. The levels of p53 are reduced after H*ras*61L transduction, and remain reduced which suggested that p53 is non-functional in the epithelial tumors, as demonstrated by absence of doxorubicin-induced G1 arrest.

Our summary of karyotypic changes involved in mammary cell transformation is outlined in Figure 6. Normal HMEC were allowed to passage through stasis, during which time the p16 promoter was likely to have been epigenetically silenced. They were then immortalized with hTERT. Cells first acquire the +der(1)t(1;8)(p13;q13), trisomy 5, trisomy 20. Following this, a fraction of the cells undergo further rearrangement involving chromosome 1, resulting in two related cell populations, one with translocation of unidentified material to the long arm of chromosome 1 at band 1q21 and the other with a pseudoisodicentric chromosome 1 (Figure 6). We hypothesize that these cell lines demonstrating the add(1) and psu idic(1) represent the primary karyotypes of the mammary carcinoma. Further complex karyotypic evolution occurs beyond these cell lines populations.

Other *in vitro *transformation models include the Weinberg model [9], the Reddel model [11], and the Miller model [34]. The tumors generated in the Weinberg study were epithelial based on the pan-cytokeratin staining. Toouli et al. [11] did not discuss the morphology of the tumor generated in their study; however, it was presumably epithelial. To achieve mammary cell transformation, the Weinberg laboratory [9,35] used a combination of four genes (H-*ras*V12G (12×), hTERT, small t antigen, and large T antigen), and the Reddel lab, the two viral oncogenes (small t antigen, large T antigen) alone. In the Reddel study, prolonged passaging of HMEC in vitro produced malignancy, in contrast to a series of transductions. In our study, passaged immortal HMEC accumulated some cytogenetic abnormalities but did not acquire tumorigenicity. Transformation of immortal HMEC was only associated with late-passage transduction of H-*ras*. Elenbaas et al. [9] state a seven-fold excess of Ras was usually inadequate to transform their HMEC, and that it was only with twelve fold excess that transformation occurred regularly. Our study suggests that a five-fold excess of 61L mutant H-ras is sufficient for transformation. Although high levels of H-ras were associated with transformation of HMEC in the Weinberg study, other genetic changes are required as well. Only 52% of mice in the Weinberg mammary epithelial cell study developed tumors with an average latency of 52 days. This is in contrast to Weinberg's earlier results [10] in which transformed human embryonic kidney cells and human fibroblasts produced tumors in nude mice in 21 days. These mesenchymal tumors showed virtually no cytogenetic changes, suggesting a less complex etiology. Perhaps connective tissue is endowed with all the tools needed for successful tumor invasion, while epithelium must recruit surrounding stromal cells early on. One of the tumors in the Reddel study required 68 days to develop, while the other malignant line grew in 21 days. H-ras transduction was not employed. The Miller model, developed at Wayne State University, is unique because of its ability to generate premalignant breast lesions. MCF 10A cells were transfected with H-ras V12G to generate MCF10AT, which were passaged by serial xenograft transplantations. Injection of cells into nude-beige mice resulted in heterogenous histologies, including hyperplastic lesions, ductal carcinoma in situ, and squamous cell carcinoma [36]. The karyotype of MCF 10AT was very stable, with a few cytogenetic alterations occurring with progression [37]. Further xenograft passaging of MCF10AT led to the development of MCF10ACA1, which rapidly and directly formed large tumors without a premalignant phase [38]. The HMEC in our study may have been nearly or fully transformed prior to injection, as tumors rapidly formed tumors in 21 days in 100% of injected mice. The non-functionality of p53 in both the Weinberg and Reddel tumors might be due to the presence of SV40 large T antigen. The tumors studied by Miller were p53 positive, and some were estrogen receptor positive. Although no exogenous viral oncogenes were introduced into our transformed cells, p53 appears to be non-functional, based on the doxorubicin G1 arrest assay. The loss of functional p53 may promote genetic instability in the mammary cells, allowing the acquisition of the complex changes required for transformation.

## Conclusion

The combination of cytogenetic changes along with H-ras 61L overexpression led to transformation in a donor's cells hTERT immortalized HMEC and allowed us to outline a stepwise model to transformation. These cytogenetic changes, which give us clues as to the nature of the genetic changes taking place, reflect a portion of the molecular events that are proceeding in our model of mammary transformation. Other molecular events required for transformation are likely taking place through alternate means such as epigenetic changes or altered translational regulation.

## Abbreviations

BSA = bovine serum albumin; DAPI = 4', 6-Diamidino-2-phenylindole; EDTA = ethylenediaminetetraacetic acid; FISH = fluorescent in situ hybridization;

HMEC = human mammary epithelial cells; HPV = human papilloma virus; hTERT = human catalytic subunit of telomerase; SV 40 = simian virus 40; ISCN = international system for human cytogenetic nomenclature; PBS = phosphate buffered saline;

SKY = spectral karyotyping; TUNEL = terminal deoxynucleotidyl transferase mediated dUTP nick end labeling

## Competing interests

The author(s) declare that they have no competing interests.

## Authors' contributions

KR and OA carried out the tissue culture, performed the animal experiments, and drafted the manuscript. KEO, GB, and KW performed the Spectral Karyotyping. EB and SOL performed the G-band karyotyping. PP and JS performed the immunohistochemistry. JKM conceived of the study and participated in its design and coordination and helped to draft the manuscript.
